# A chimeric strain of porcine reproductive and respiratory syndrome virus 2 derived from HP-PRRSV and NADC30-like PRRSV confers cross-protection against both strains

**DOI:** 10.1186/s13567-024-01390-y

**Published:** 2024-10-07

**Authors:** Yang Li, Yumiao Wang, Xiuxiu Pei, Shao Chen, Yang Jing, Yongshuai Wu, Zhiqian Ma, Zhiwei Li, Zifang Zheng, Yingtong Feng, Lele Xu, Xiao Liu, Xuyang Guo, Haixue Zheng, Shuqi Xiao

**Affiliations:** 1 State Key Laboratory for Animal Disease Control and Prevention, College of Veterinary Medicine, Lanzhou University, Lanzhou Veterinary Research Institute, Chinese Academy of Agricultural Sciences, Lanzhou, 730000 China; 2 College of Veterinary Medicine, Northwest A&F University, Yangling, Xianyang, 712100 China

**Keywords:** Porcine reproductive and respiratory syndrome (PRRS), NADC30-like PRRSV, HP-PRRSV, cross-protection, infectious clone, genetically engineered vaccine, chimeric PRRSV

## Abstract

Porcine reproductive and respiratory syndrome (PRRS) is one of the most significant swine viral infectious diseases worldwide. Vaccination is a key strategy for the control and prevention of PRRS. At present, the NADC30-like PRRSV strain has become the predominant epidemic strain in China, superseding the HP-PRRSV strain. The existing commercial vaccines offer substantial protection against HP-PRRSV, but their efficacy against NADC30-like PRRSV is limited. The development of a novel vaccine that can provide valuable cross-protection against both NADC30-like PRRSV and HP-PRRSV is highly important. In this study, an infectious clone of a commercial MLV vaccine strain, GD (HP-PRRSV), was first generated (named rGD). A recombinant chimeric PRRSV strain, rGD-SX-5U2, was subsequently constructed by using rGD as a backbone and embedding several dominant immune genes, including the NSP2, ORF5, ORF6, and ORF7 genes, from an NADC30-like PRRSV isolate. In vitro experiments demonstrated that chimeric PRRSV rGD-SX-5U2 exhibited high tropism for MARC-145 cells, which is of paramount importance in the production of PRRSV vaccines. Moreover, subsequent in vivo inoculation and challenge experiments demonstrated that rGD-SX-5U2 confers cross-protection against both HP-PRRSV and NADC30-like PRRSV, including an improvement in ADG levels and a reduction in viremia and lung tissue lesions. In conclusion, our research demonstrated that the chimeric PRRSV strain rGD-SX-5U2 is a novel approach that can provide broad-spectrum protection against both HP-PRRSV and NADC30-like PRRSV. This may be a significant improvement over previous MLV vaccinations.

## Introduction

Porcine reproductive and respiratory syndrome (PRRS) is one of the most significant viral diseases that has economically decimated the swine industry worldwide since its emergence in the 1980s [1–3]. Porcine reproductive and respiratory syndrome virus (PRRSV) is a small, enveloped, single-stranded positive-sense RNA virus that belongs to the genus *Betaarterivirus*, family *Arteriviridae*, and order *Nidovirales* according to the latest classification [4–7]. PRRSV is one of the most rapidly evolving RNA viruses. The calculated rate of nucleotide substitutions is approximately 4.7 × 10^–2^ to 9.8 × 10^–2^/site/year [8]. PRRSV can be divided into two genotypes: PRRSV-1 (genotype 1, European) and PRRSV-2 (genotype 2, North American), which exhibit approximately 60% nucleotide sequence identity [1, 9, 10]. Additionally, although PRRSV-1 has been observed in China, PRRSV-2 has been the predominant epidemic strain in recent years [11–14].

The complete genome of PRRSV is approximately 15 kb, and it contains at least 10 open reading frames (ORFs). The ORF1a and ORF1b genes encode nonstructural proteins (NSP1 ~ NSP12), which are associated with the processing of viral polyproteins, genome replication, and transcription [15–17]. ORF2a, ORF2b, ORF3, ORF4, ORF5, ORF5a, ORF6, and ORF7 encode eight viral structural proteins (Gp2a, E, Gp3, Gp4, Gp5, Gp5a, M, and N) that are essential for the assembly of the virion [18–21]. PRRSV was first isolated in China in 1996 and has been spreading for almost 30 years since then. In 2006, a highly pathogenic PRRSV (HP-PRRSV) swept the pig industry throughout China, resulting in significant economic losses. In 2013, NADC30-like PRRSV emerged in China and rapidly spread throughout the country. NADC30-like PRRSV is less pathogenic than HP-PRRSV, but it has high variability and is easily recombined with other PRRSV strains to form new isolates with varied genomic characteristics, cell tropisms, and pathogenicity [22–24]. Currently, NADC30-like PRRSV has become the dominant epidemic strain in China [25–27].

Vaccination represents a pivotal strategy for the control and prevention of PRRS. Currently, nine commercial vaccines are widely utilized in China, including CH1a/CH1R, RespPRRS MLV, R98, TJM-F92, HuN4-F112, GDr180, JXA1-R, and PC. These vaccines are divided into two categories: classical PRRSV and HP-PRRSV [22, 26]. These vaccines have made significant contributions to the prevention and control of PRRSV, particularly in the context of homologous PRRSV strains. Nevertheless, owing to the high genetic, antigenic, and pathogenic heterogeneity of different PRRSV strains, the protection provided by these commercial vaccines against heterologous NADC30-like PRRSV strains is not optimal [28, 29]. Therefore, developing a vaccine that offers broad protection against PRRS is highly important. Porcine alveolar macrophages (PAMs) are the main target cells of PRRSV, but they are difficult to isolate and cannot be passaged. MARC-145 cells have proven to be valuable tools for the development and production of PRRSV vaccines. Indeed, it is estimated that almost all commercial PRRSV vaccines are produced on the basis of MARC-145 cells [30–32]. Nevertheless, numerous investigations have demonstrated that an increasing number of NADC30-like PRRSV isolates alter the tropism of MARC-145 cells, significantly impeding the development of NADC30-like PRRSV vaccines [22, 33–35].

In the present study, we attempt to broaden the protective coverage of existing PRRS MLV vaccines by constructing a chimeric strain of HP-PRRSV (the MLV vaccine strain) and NADC30-like PRRSV. An infectious clone of a commercial MLV vaccine strain, GD (HP-PRRSV), was first generated (named rGD) as we previously described [36, 37]. A recombinant chimeric PRRSV strain, rGD-SX-5U2, was subsequently constructed by using rGD as a backbone and embedding several dominant immune genes, including the NSP2, ORF5, ORF6, and ORF7 genes of a NADC30-like PRRSV. rGD-SX-5U2 displays tropism for MARC-145 cells. More importantly, further experiments demonstrated that chimeric rGD-SX-5U2 confers cross-protection against both HP-PRRSV and NADC30-like PRRSV in piglets. Our research revealed a new approach that can generate broad-spectrum protection against both HP-PRRSV and NADC30-like PRRSV, which may be a significant improvement over previous MLV vaccinations.

## Materials and methods

### Viruses, cells, antibodies, and plasmids

The PRRSV strains used in this study, including HP-PRRSV SD-YL1712 (MT708500), NADC30-like PRRSV SX-YL1806 (OR208175), and a modified live virus (MLV) vaccine PRRSV strain GD, were all stored in our laboratories. MARC-145 cells (an African green monkey kidney cell line) and BHK-21 (baby hamster kidney 21) cells were maintained in Dulbecco’s modified Eagle’s medium (DMEM; Thermo Fisher, USA) supplemented with 10% foetal bovine serum (FBS; Gibco, USA). PAMs were harvested from the lungs of healthy 6-week-old piglets as previously described and cultured in RPMI-1640 medium supplemented with 10% FBS [38]. A monoclonal antibody (6D10) against the PRRSV N protein was prepared and stored in our laboratory. The bacterial artificial chromosome (BAC) vector pBeloBAC11 used in this study was stored in our laboratories.

### Strategies for constructing full-length cDNA clones of the PRRSV MLV strain GD

**Figure 1:**
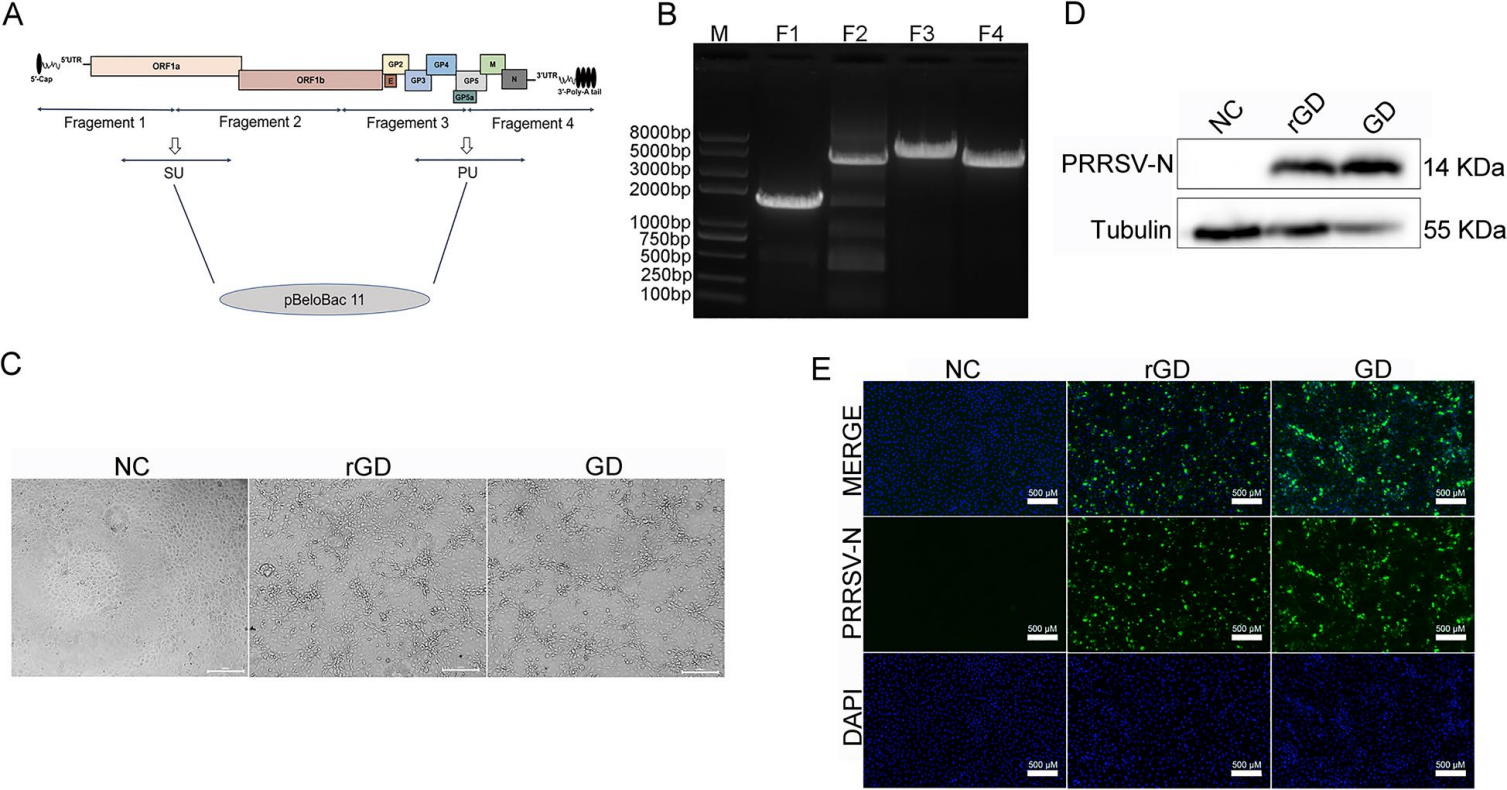
**Design, construction and rescue of the PRRSV MLV strain rGD infectious clone**. **A** Strategy used for constructing the full-length PRRSV MLV strain. **B** Amplification of four fragments of the whole genome of the PRRSV MLV strain GD by PCR. **C** Cytopathic effect (CPE) induced by rescued PRRSV rGD in MARC-145 cells. MARC-145 cells were infected with supernatants removed from BHK-21 cells transfected with recombinant pBAC-GD plasmids. Wild-type PRRSV GD was used as a positive control. **D** The presence of rescued PRRSV rGD was identified by western blotting in MARC-145 cells. **E** Immunofluorescence assay (IFA) was used to detect the rescued PRRSV rGD strain in MARC-145 cells. Wild-type PRRSV GD was used as a positive control, and DMEM was used as a negative control.

Accurate, complete genome sequences of the PRRSV MLV strain GD were obtained by RT-PCR and 5’- and 3’-RACE (TAKARA, Japan). The whole genome was divided into four fragments, F1, F2, F3, and F4; these four fragments were then amplified by PCR using PrimeSTAR GXL DNA Polymerase (TAKARA, Japan) with the primers listed in Table 1. Subsequently, an overlap PCR including fragments F1 and F2 or F3 and F4 was performed with primers F1-F and F2-R or F3-F and F4-R to generate a chimeric Fragment SU or PU. The modified bacterial artificial chromosome vector pBeloBAC11 (pBAC) stored in our laboratory was subsequently linearized by two restriction endonucleases (NEB, USA), *Sfi* I and *Rsr* II. Furthermore, fragments SU and PU were inserted into linearized pBAC vectors to assemble the infectious cloning plasmid pBAC-GD by homologous reorganization operations (Vazyme, China), which contained the full-length PRRSV GD strain. The recombinant pBAC-GD plasmids were prepared using QIAfilter Plasmid Kits (QIAGEN, Germany) and verified by resequencing. The design of the recombinant PRRSV infection clone pBAC-GD is shown in Figure 1A .

**Table 1:** **Primers used for construction of the PRRSV MLV strain rGD or chimeric PRRSV rGD-SX-5U2 infectious clones**

Primer	Sequence	Product size(bp)
F1-F	*AGTGAACCGTGGCCCGGGCGGCC*ATGACGTATAGGTGTTGGCTCT	1937
F1-R	*ACTGCCGGACATTTCCGCCAGGGCTGCCGGGA*TGACACTACTAGGC	
F2-F	*TCCCGGCAGCCCTGGCGGAAATGTCCGGCAGT*TTTGGTTGTTCAACACCT	3954
F2-R	*CTTCCAGTTCGGGTT*TGGCAGCAAGCAGGGCACAA	
F3-F	*AACCCGAACTGGAAG*GAGGCCTTTCCACAGTTCAACT	5400
F3-R	*CGGCCTTCAAGTTGAAAATA*GGCCGTCTTGTCTTTCCATAC	
F4-F	*ATTTTCAACTTGAAGGCCG*CCATTTTACCTGGTATCAACTTGCAAG	3889
F4-R	CGGATGCCCAGGTCGGACCGCGAGGAGGTGGAGATGCCATGCCGACCCTTTTTTTTTTTTTTTTTTTTTTTTT*AATTTCGGCCGCATGGTTCTCGCCAATT*	
Fragement A-F	*GGCAATTTGAATGTTCA*AGTATGTTGGGGAAATGCTTGACCG	1516
Fragement A-R	*GATGTCTCAAGAATGTCAGC*CCATCATGCTGAAGGTGGCGTTGTG	
Fragement B-F	*CTGACTAAGGAGCAGTGTTTAAAC*TGCTAGCCGCCAGCGG	6113
Fragement B-R	*TGAACATTCAAATTGCC*AGTAGGATGGCAAAAAGACAGGCTAAA	
Fragement C-F	*GCTGACATTCTTGAGACATC*CTGGTGTTTGAATTGGAAGAATGC	218
Fragement C-R	ATGCCCAGGTCGGACCGCGAGGAGGTGGAGAT	
Fragement E-F	*CATAAGTGGTACGGTGCTGG*GAAGAGAGCAAGAAGAGCA	3222
Fragement E-R	*GAGGTGTGGGCCTCCTCCTGAA*GACTTGGAGATCTGCCT	
Fragement F-F	*ACCGTGGCCCGGGCGGCC*ATGACGTATAGGTGTTGGCTCT	1361
Fragement F-R	*CCAGCACCGTACCACTTATG*ACTGCCAAACCG	
Fragement G-F	*TTCAGGAGGAGGCCCACACCTC*ATTGCTGCC	2779
Fragement G-R	*GTTTAAACACTGCTCCTTAGTCAG*GCCTTGGAGTTTGTC	

Homologous arm sequences in italics and restriction site sequences in underscores.

### Transfection and rescue of the recombinant virus rGD

The recombinant PRRSV was rescued according to the methods described in a previous report [36]. Briefly, BHK-21 cells were grown to 70% confluence in a six-well plate, and 2.5 μg of the recombinant pBAC-GD plasmid was transfected into BHK-21 cells with PEI transfection reagent (Thermo Fisher, USA) following the manufacturer’s protocol to rescue infectious PRRSV. After 12 h, the supernatant was removed, and the mixture was washed 3 times with PBS (Beyotime, China), after which the medium was replaced with DMEM containing 10% FBS. Moreover, MARC-145 cells were cultured to 70% confluence in a six-well plate, and the supernatants removed from BHK-21 cells were added to MARC-145 cells. The CPE was monitored daily after the transfer. After 3 days, the supernatants were collected and used to infect fresh MARC-145 cells to propagate the rescued virus. The rescued virus was named PRRSV rGD and was used for further analysis after three successive generations of propagation.

### Construction and rescue of a chimeric PRRSV rGD-SX-5U2

**Figure 2:**
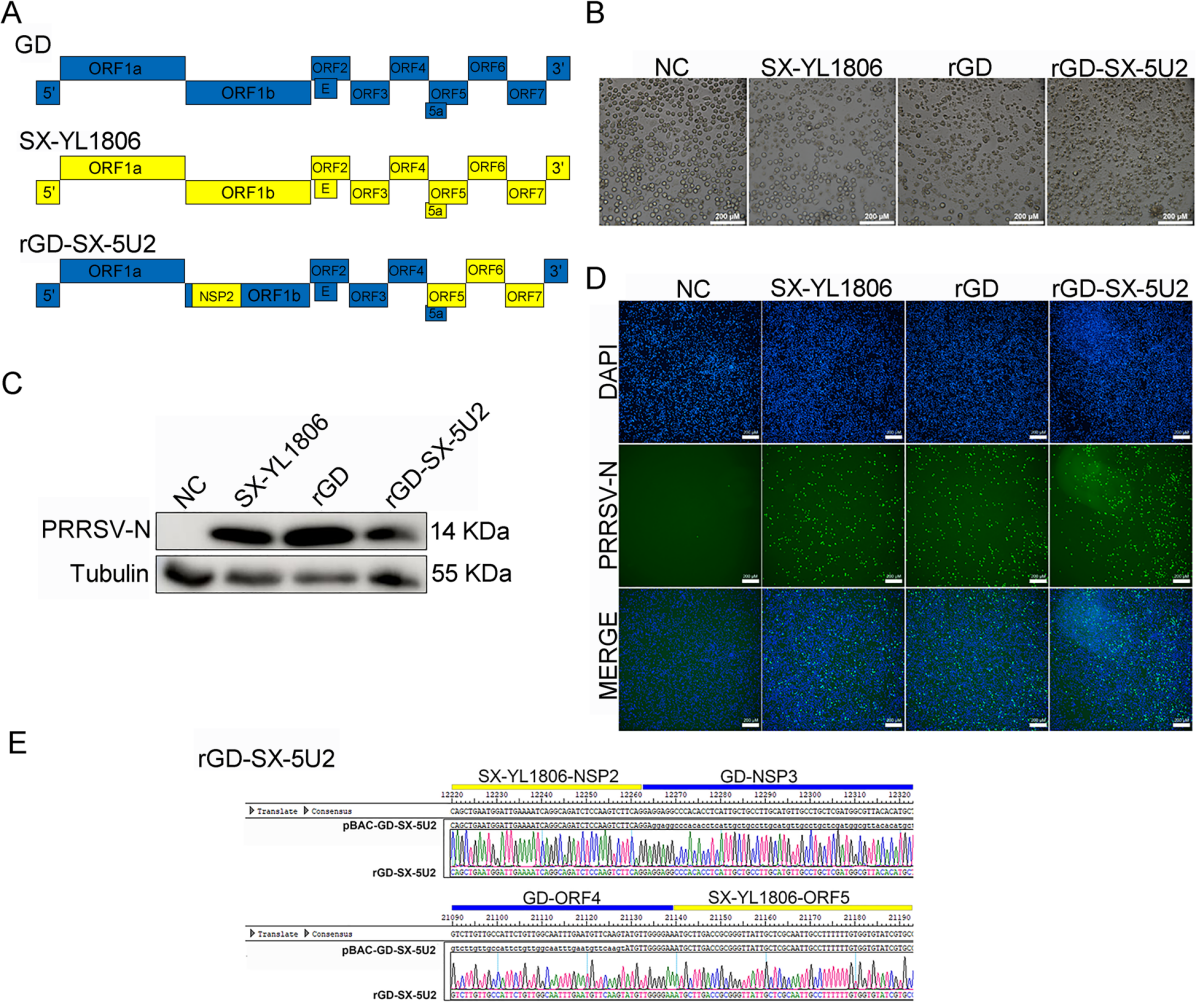
**Design and rescue of the chimeric PRRSV strain rGD-SX-5U2 infectious clone**. **A** Strategy used for constructing the full-length chimeric PRRSV strain rGD-SX-5U2. rGD-SX-5U2 originated from HP-PRRSV and NADC30-like PRRSV by using the rGD as a backbone and embedding some genes, including the NSP2, ORF5, ORF6, and ORF7 genes from the NADC30-like PRRSV isolate SX-YL1806. **B** CPE induced by rescued chimeric PRRSV rGD-SX-5U2 in PAMs. Wild-type PRRSV GD and SX-YL1806 were used as positive controls. **C** The presence of rescued chimeric PRRSV rGD-SX-5U2 in PAMs was identified by western blotting. PAMs were infected with PRRSV (MOI = 0.1) and harvested at 36 hpi. **D** IFA was also used to detect the rescued chimeric PRRSV rGD-SX-5U2 strain in PAMs. PAMs were infected with PRRSV rGD-SX-5U2 (MOI = 0.1) and harvested at 36 hpi. **E** Sequencing results of several key chimeric locations of PRRSV rGD-SX-5U2. The whole genome of the chimeric strain rGD-SX-5U2 was extracted and sequenced by a third-party company, focusing on the sequence of the chimeric parts of this chimeric PRRSV strain.

To generate an infectious clone carrying the HP-PRRSV and NADC30-like PRRSV sequences at the same time, the full-genome cDNA clone of the HP-PRRSV MLV strain GD was used as the backbone, and its ORF5, ORF6, ORF7, and NSP2 genes were replaced with the corresponding genes of the NADC30-like PRRSV SX-YL1806 (Table 1). Briefly, the ORF5, ORF6, and ORF7 genes of PRRSV SX-YL1806 were amplified with the primers Fragment A-F and Fragment A-R, the amplified product of which was named Fragment A. Then, the pBAC-GD infectious clone was amplified with the primers Fragment B-F and Fragment B-R, Fragment C-F and Fragment C-R to produce two fragments, named Fragment B and Fragment C. Subsequently, overlap PCR, including Fragment A, Fragment B and Fragment C, was performed with the primers Fragment B-F and Fragment C-R to generate a chimeric Fragment D containing ORF5, ORF6, and ORF7 genes of the NADC30-like PRRSV SX–YL1807 and other genes of the HP-PRRSV GD. Then, the pBAC-GD plasmid was linearized by using two restriction endonucleases (NEB, USA), *Pem* I and *Rsr* II, and Fragment D was ligated into it to generate the chimeric clone vector pBAC-GD-SX-5U. Similarly, the NSP2 gene of NADC30-like PRRSV SX-YL1806 was amplified with the primers Fragment E–F and Fragment E-R to produce a fragment named Fragment E. Furthermore, some genes of PRRSV GD were amplified with the primers Fragment F-F and Fragment F-R, Fragment G-F and Fragment G–R to produce two fragments, named Fragment F and Fragment G. Subsequently, overlap PCR, including Fragment E, Fragment F and Fragment G, was performed with the primers Fragment F-F and Fragment G-R to generate a chimeric Fragment H containing the NSP2 gene of the NADC30-like PRRSV SX-YL1807 and other genes of the HP-PRRSV GD. Then, the pBAC-GD-SX-5U plasmid was linearized by using two restriction endonucleases (NEB, USA), *Sfi* I and *Pem* I, and Fragment H was ligated into it to generate the chimeric clone vector pBAC-GD-SX-5U2. The design of the recombinant PRRSV infection clone pBAC-GD-SX-5U2 is shown in Figure 2A. The recombinant plasmid was identified by sequencing.

The chimeric PRRSV was rescued as described previously with minor modifications. The recombinant plasmid pBAC-GD-SX-5U2 was transfected into BHK-21 cells using PEI transfection reagent following the manufacturer’s protocol. Then, the culture supernatant collected 48 h post-transfection (hpt) was serially passaged into PAMs. The recovery of chimeric infectious PRRSV rGD-SX-5U2 was confirmed by an indirect immunofluorescence assay (IFA) and a western blotting assay (WB) in which a monoclonal antibody (6D10) against the PRRSV N protein was used. An anti-mouse IgG antibody labelled with Alexa Fluor 488 (obtained from Abways Technology) was used as the secondary antibody. Moreover, the rescued PRRSV was named rGD-SX-5U2 and confirmed by sequencing.

### Immunofluorescence assays

Immunofluorescence assays (IFA) were performed as described previously [39] with the following modifications: MARC-145 cells or PAMs were cultured on glass cover slips in a 24-well plate and infected with rGD-SX-5U2 (5^th^ passage), GD, rGD or SX-YL1806 at a multiplicity of infection (MOI) of 1.0 for 36 h. The samples were collected, washed three times with PBS, fixed with 4% paraformaldehyde (Solarbio, China) for 15 min at 37 ℃, and then permeabilized for 10 min with 0.25% Triton X-100 (Solarbio, China). The cells were subsequently incubated with 1% BSA (Solarbio, China) for 30 min at 37 °C to block nonspecific binding. The cells were subsequently incubated with a monoclonal antibody (6D10) against the PRRSV N protein for 1 h at 37 ℃. After that, the cells were washed three times with PBS and further incubated with anti-mouse IgG antibodies labelled with Alexa Fluor 488 (Jackson, USA) for 1 h at 37 ℃. Finally, the samples were washed three times with PBS and stained with 4-6-diamidino-2-phenylindole (DAPI, Beyotime, China) for 7 min at 37 ℃. The samples were then visualized with a fluorescence microscope. Mock-infected cells were used as a negative control. Other PRRSV-infected cells were used as positive controls.

### Western blotting

Western blotting was performed as described previously [40] with minor modifications: MARC-145 cells or PAMs were infected with rGD-SX-5U2 (5^th^ passage), GD, rGD or SX-YL1806 at an MOI of 1.0. When 60% of the cells showed CPEs, the infected MARC-145 cells or PAMs were harvested and lysed in RIPA buffer (Solarbio, China) containing protease inhibitors (Solarbio, China). The lysates were separated by 12% SDS‒PAGE and transferred onto polyvinylidene difluoride (PVDF) membranes. The membranes were blocked with 5% skimmed milk in PBST for 1 h at room temperature and then incubated with the anti-β-Tubulin antibody (Sigma‒Aldrich, USA) or 6D10 overnight at 4 °C. The membranes were washed with PBST and then incubated with peroxidase-conjugated goat anti-mouse IgG (Thermo Fisher, USA) for 1 h at room temperature. After washing, the target proteins were detected with an enhanced chemiluminescence (ECL) kit (Beyotime, China). Cellular proteins from mock-infected MARC-145 cells were used as a negative control. Other PRRSV-infected cells were used as positive controls.

### Real-time qPCR assay

RT-qPCR was performed as described previously with minor modifications [41]. The cells or serum samples were washed with PBS and lysed in RNAiso Plus (TaKaRa, Japan), and reverse transcription was performed using a PrimeScript RT reagent kit (Vazyme, China) according to the manufacturer’s instructions. RT-qPCR was performed with the ChamQ SYBR qPCR Master Mix (Vazyme, China) according to the manufacturer’s instructions. Three replicates were used, and a recombinant plasmid containing the ORF7 gene of PRRSV was used to construct a standard curve. This plasmid was tenfold serially diluted to obtain concentrations ranging from 10^–2^ to 10^–10^ copies/μL. The number of PRRSV RNA copies was calculated according to the standard curve.

### Determination of growth curves for chimeric PRRSV rGD-SX-5U2

NADC30-like PRRSV SX-YL1806 lost tropism to MARC-145 cells. To determine the infection ability of the chimeric PRRSV strain rGD-SX-5U2 in MARC-145 cells, growth curves were generated as described previously with minor modifications [28]. MARC-145 cells were infected with rGD-SX-5U2 or rGD at an MOI of 0.1, and the growth curves of rGD-SX-5U2 and the parental virus rGD were compared. Briefly, 2 h after virus adsorption, MARC-145 cells were washed with PBS three times and incubated in DMEM supplemented with 3% FBS at 37 °C and 5% CO_2_. The supernatants were collected at the indicated time points (0, 12, 24, 36, 48, 60, and 72 hpi), and viral titres were calculated via a TCID_50_ assay (calculated via the Reed‒Muench method).

### Plaque assays

Plaque assays were performed as described previously [36] with moderate modifications. MARC-145 cell monolayers in 6-well plates were infected with 2 mL of a tenfold serially diluted rescued PRRSV rGD-SX-5U2. Two hours after virus adsorption, the cell monolayers were washed with PBS three times and then overlaid with 1% low-melting agarose (Yeasen, China) in DMEM supplemented with 3% FBS. At 4 dpv, the plaques were visualized with neutral red dye (Solarbio, China). PRRSV SX-YL1806 and rGD were used as controls.

### Animal inoculation and challenge study

To evaluate the protective effect of chimeric PRRSV rGD-SX-5U2 against HP-PRRSV and NADC30-like PRRSV, pig inoculation and challenge studies were performed (Table 2). Twenty-three 4-week-old PRRSV-free piglets were randomly divided into five groups (five piglets in the infected group and three piglets in the control group). Piglets in groups A and B were intramuscularly inoculated with 2 mL of DMEM at 0 dpv. Piglets in groups C and D were intramuscularly inoculated with 2 mL of 10^5.0^ TCID_50_/mL rGD-SX-5U2 (5^th^ passage) at 0 dpv. Piglets in group E were inoculated with DMEM at 0 dpv to serve as the control. At 42 days postvaccination (dpv), piglets in groups A and C were challenged with 3 mL of 10^5.0^ TCID_50_/mL HP-PRRSV SD-YL1712, and piglets in groups B and D were challenged with 3 mL of 10^5.0^ TCID_50_/mL NADC30-like PRRSV SX-YL1806. Piglets in group E were challenged with DMEM to serve as the control. All the groups were fed separately in a biosafety room.

**Table 2:** **Vaccination and challenge strategies**

Group	Number	Vaccination (0 dpv)	Challenge (42 dpv)	Dose
A (NC-HP)	5	DMEM	HP-PRRSV	4 × 10^5^TCID_50_
B (NC-DC30)	5	DMEM	NADC30-like PRRSV	
C (5U2-HP)	5	rGD-SX-5U2	HP-PRRSV	
D (5U2-DC30)	5	rGD-SX-5U2	NADC30-like PRRSV	
E (NC)	3	DMEM	DMEM	

The behaviour of each piglet was observed daily for any unusual clinical condition, such as coughing, sneezing, depression, or diarrhoea. The rectal temperature of each piglet was recorded daily during the entire experiment. The growth performance of each piglet was recorded by its body weight gain rate, which was calculated by recording the body weight weekly. The serum samples of pigs in each group were collected at 0, 4, 7, 11, 14, 21, 28, 35, 42, 49, 56, and 63 dpv to measure the virus load and detect antibodies. A real-time RT-PCR assay was conducted to evaluate the dynamics of viremia. PRRSV-specific antibodies were detected by the Porcine Reproductive and Respiratory Syndrome Virus AB Elisa Kit (JNT, China) according to the manufacturer’s instructions, and the threshold for seroconversion was set at an S/P (sample-to-positive) ratio of 0.4. The sera collected at 42 dpv were subjected to a virus neutralization test.

Additionally, all the piglets were euthanized for autopsy at 63 dpv, and lung tissue samples were taken from each piglet. A section of the lung tissue was subjected to RT-qPCR to determine the viral load, and another portion was preserved in 4% paraformaldehyde for use in the immunohistochemical and histological analyses performed by Shaanxi Yike Biotechnology Service Co. Ltd. (China). Macroscopic lesions and scores of the lungs were estimated as previously discussed and were based on assigning a number to each lobe to reflect the approximate percentage of the overall lung represented by that lobe. On the basis of the extent and magnitude of interstitial pneumonia, the microscopic lesions and scores of the lung tissues were estimated as follows: 0, no lesion; 1, mild/focal; 2, moderate/multifocal; 3, moderate/diffuse (alveolar wall accounting for greater than 50% of the measured section); and 4, severe/diffuse (alveolar wall accounting for greater than 75% of the measured section). Macroscopic and microscopic lung lesions were evaluated by three pathologists who were blinded to the test [28, 42, 43].

### Serum virus neutralization (SVN) assay

Serum virus neutralization assays were performed as described previously with moderate modifications [28]. The serum samples collected at 42 dpv were first heat inactivated at 56 ℃ and then diluted and incubated with 200 TCID_50_ of viruses containing HP-PRRSV SD-YL1712 or NADC30-like PRRSV SX-YL1806 in medium supplemented with 3% FBS for 1 h. Then, the virus‒antibody mixture was transferred to a 96-well plate of confluent PAMs and incubated until the CPE was detected. Then, an IFA was carried out to identify the CPE induced by PRRSV. The absence of CPE at a 1:8 dilution was considered positive for the presence of PRRSV neutralization [18, 26].

### Statistical analysis

Data analysis in this study was performed with GraphPad Prism 6 software (La Jolla, CA, USA) via one-way or two-way ANOVA followed by Tukey’s t test. Accordingly, a *P* value of < 0.05 was considered to indicate a significant difference.

## Results

### Construction and rescue of an infectious PRRSV MLV strain rGD from a cDNA clone

The HP-PRRSV MLV strain GD was divided into 4 fragments (designated F1–F4) to generate a construct spanning the entire genome of PRRSV GD (Figure 1A). These four fragments were amplified and inserted into linearized pBAC vectors to assemble the infectious cloning plasmid pBAC-GD by homologous reorganization operations. The pBAC-GD plasmid was identified using four pairs of primers that can amplify fragments F1, F2, F3, and F4 (Figure 1B). The pBAC-GD plasmid was transfected into BHK21 cells at a confluence of approximately 70% in six-well plates, and then the culture supernatant collected at 48 hpt was removed from BHK-21 cells and serially passaged in MARC-145 cells. The wild-type PRRSV GD was used as a positive control. CPE was monitored daily, and after 48 h, CPE appeared in the cells transfected with pBAC-GD and infected with the wild-type PRRSV GD (rescued strains named PRRSV rGD) (Figure 1C). The PRRSV rGD gene was identified by western blotting (Figure 1D) and IFA (Figure 1E). using a monoclonal antibody (6D10) against the PRRSV N protein. Thus, the recombinant PRRSV rGD was successfully rescued.

### Construction and rescue of the chimeric PRRSV rGD-SX-5U2 from the cDNA clone

The full-genome cDNA clone of PRRSV GD was used as the backbone, and its ORF5, ORF6, ORF7, and NSP2 genes were replaced with the corresponding genes of NADC30-like PRRSV SX-YL1806 (Figure 2A). and the chimeric plasmid named pBAC-GD-SX-5U2. Similarly, pBAC-GD-SX-5U2 was transfected into BHK21 cells at a confluence of approximately 70% in six-well plates, the culture supernatant collected at 48 hpt was removed from BHK-21 cells and serially passaged in PAMs, and the CPE was monitored daily. Rescued PRRSV rGD and wild-type PRRSV SX-YL1806 were used as positive controls. CPE appeared in the cells infected with SX-YL1806, rGD, and the supernatant of pBAC-GD-SX-5U2 at 48 hpt, whereas no CPE was observed in the NC group (Figure 2B). The rescued PRRSV strain, rGD-SX-5U2, was further identified by western blot and IFA assays using a monoclonal antibody (6D10) against the PRRSV-N protein (Figures 2C, D). Moreover, Sanger sequencing revealed that the NSP2, ORF5, ORF6, and ORF7 genes of the chimeric PRRSV rGD-SX-5U2 originated from the NADC30-like PRRSV SX-YL1806 gene, whereas the other genes originated from the HP-PRRSV GD gene (Figure 2E). Thus, these results showed that the chimeric PRRSV rGD-SX-5U2 was successfully rescued.

### Chimeric PRRSV rGD-SX-5U2 maintains cell tropism to MARC-145 cells

**Figure 3:**
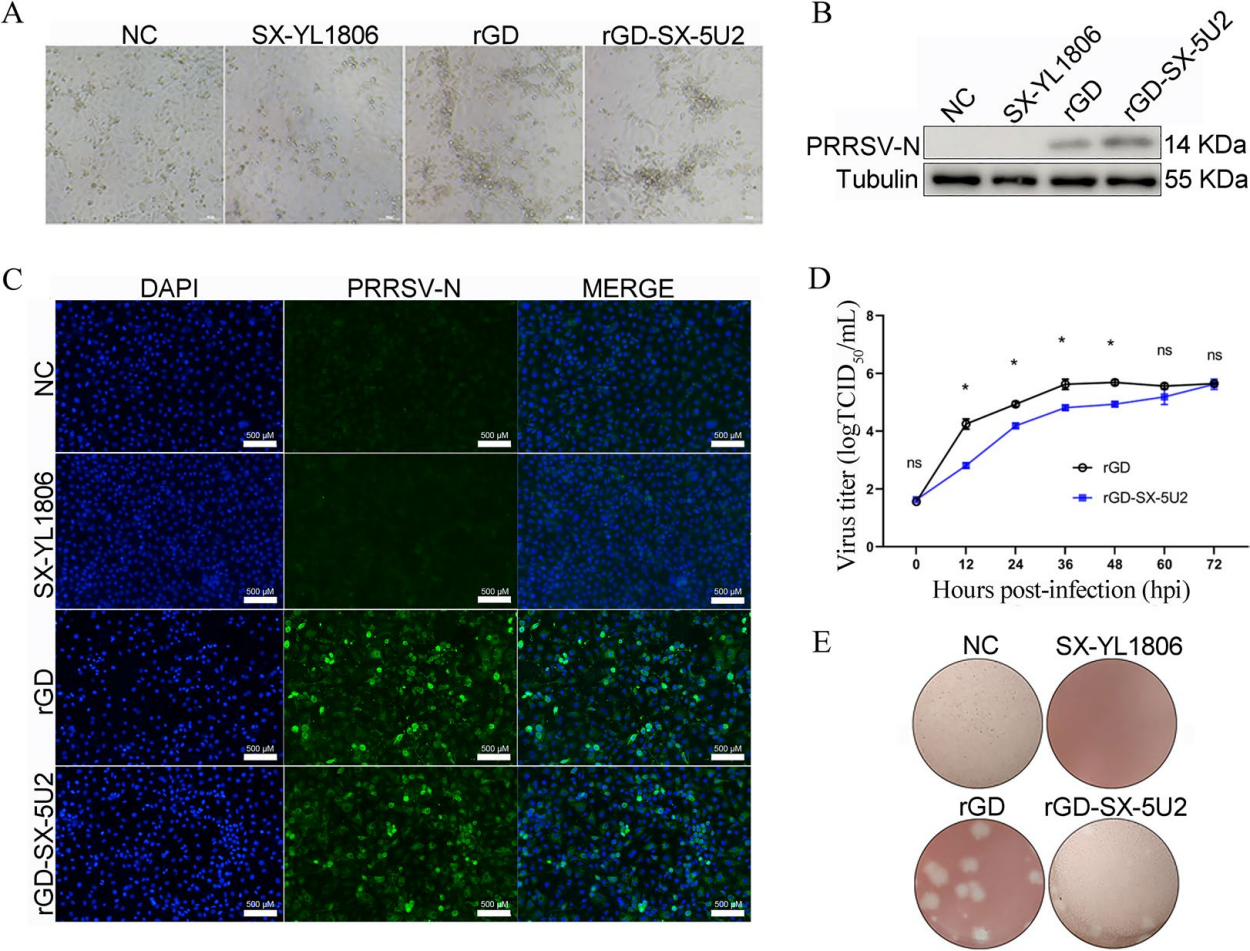
**Chimeric PRRSV rGD-SX-5U2 maintains cell tropism to MARC-145 cells**. **A** CPE induced by rescued chimeric PRRSV rGD-SX-5U2 in MARC-145 cells. MARC-145 cells infected with the rescued chimeric PRRSV rGD-SX-5U2, PRRSV rGD (MARC-145 cells infected) or NADC30-like PRRSV SX-YL1806 (MARC-145 cells not infected) were used as controls. **B** The infectivity of the rescued chimeric PRRSV strain rGD-SX-5U2 against MARC-145 cells was identified by western blotting. MARC-145 cells were infected with rGD-SX-5U2 (MOI = 0.1) and harvested at 36 dpv. **C** IFA was used to verify the infectivity of rGD-SX-5U2 in MARC-145 cells. **D** The virus titre of the rescued chimeric PRRSV rGD-SX-5U2 was determined in MARC-145 cells. MARC-145 cells were infected with rGD-SX-5U2 or rGD (MOI = 0.1). **E** Representative images of the plaque morphologies of rGD-SX-5U2 in MARC-145 cells; PRRSV rGD and SX-YL1806 were used as controls.

Our previous study revealed that NADC30-like PRRSV SX-YL1806 lost cell tropism to MARC-145 cells, but the mechanism has not been elucidated [28]. The chimeric PRRSV rGD-SX-5U2 originated from HP-PRRSV GD and NADC30-like PRRSV SX-YL1806. Therefore, it was necessary to study the infectivity of the chimeric strain rGD-SX-5U2 in MARC-145 cells. MARC-145 cells were infected with rGD-SX-5U2 at an MOI of 0.1, and the CPE was monitored daily. PRRSV rGD and SX-YL1806 were used as controls. Typical CPEs, such as the disintegration of cells induced by the PRRSV rGD-SX-5U2 and rGD strains, were observed 48 h postinfection, whereas no CPEs were observed in the SX-YL1806 or NC groups (Figure 3A). To further identify PRRSV rGD-SX-5U2 infection in MARC-145 cells, IFA and western blot assays were carried out. The results revealed that the PRRSV-N protein could be detected in MARC-145 cells inoculated with the PRRSV rGD-SX-5U2 and rGD strains by western blot analysis (Figure 3B). In addition, obvious positive fluorescent signals were detected in MARC-145 cells inoculated with the rGD-SX-5U2 and rGD strains by IFA, whereas no signals were detected in the SX-YL1806 or NC group (Figure 3C). Furthermore, the multiple-step growth curves revealed that the viral titre of the chimeric PRRSV rGD-SX-5U2 in MARC-145 cells increased over time, peaking at 10^5.625^/mL at 72 hpi. Before 72 hpi, the replication rate of the rGD-SX-5U2 strain was slightly lower than that of the rGD strain, but there was no significant difference between these two strains at 60 hpi (Figure 3D). The results of the plaque assay revealed that chimeric PRRSV rGD-SX-5U2 produced obvious plaques after infection with MARC-145 cells, whereas no obvious plaques were observed in the NC group (Figure 3E). These results indicated that the rescued chimeric PRRSV rGD-SX-5U2 is infectious in vitro and has good tropism for MARC-145 cells.

### Chimeric PRRSV rGD-SX-5U2 confers cross-protection against both HP-PRRSV and NADC30-like PRRSV

**Figure 4:**
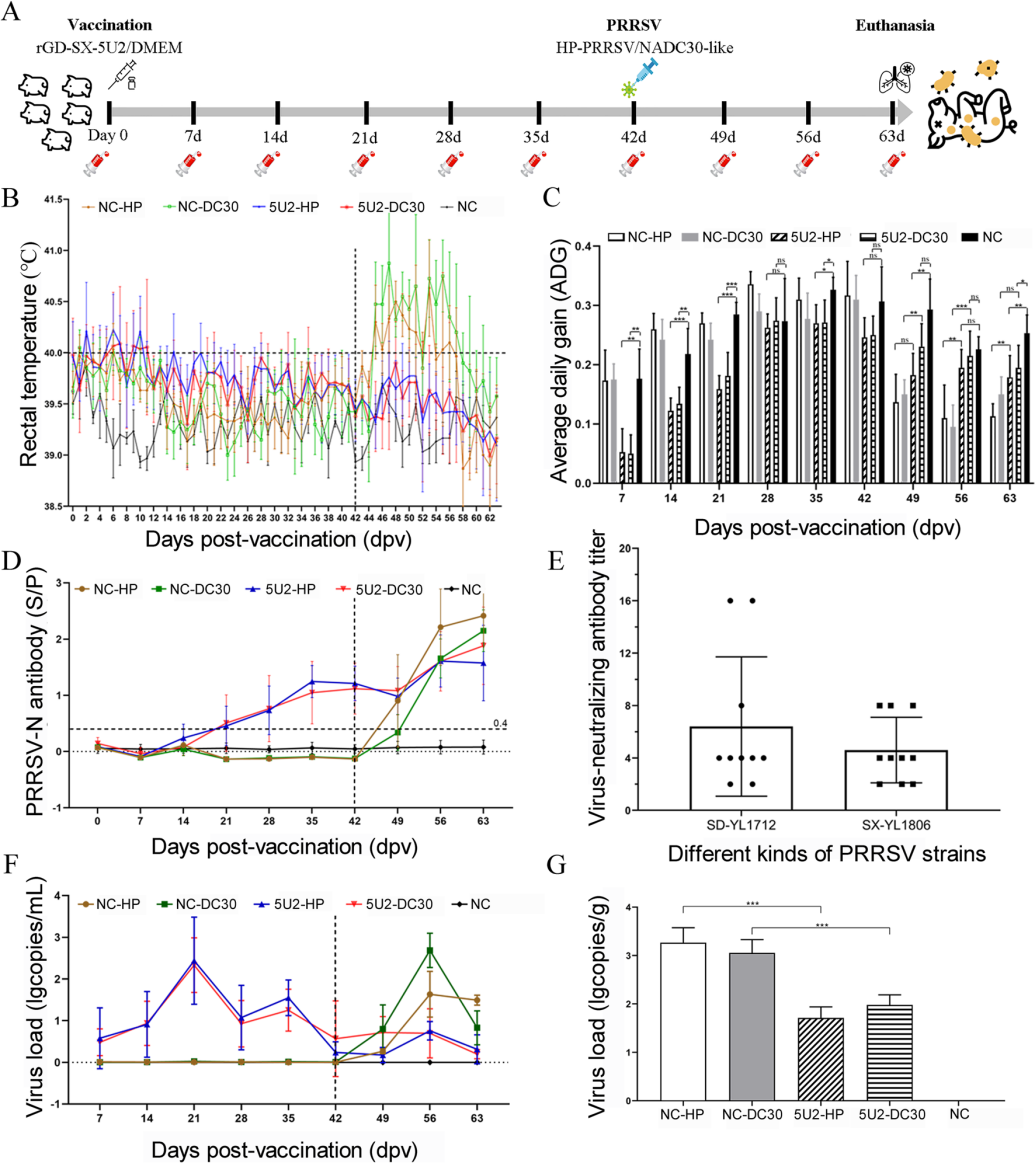
**Vaccination and challenge strategies**. **A** At 0 dpv, 4-week-old healthy pigs were inoculated with chimeric PRRSV rGD-SX-5U2 or with DMEM as a mock vaccination control. Then, the piglets immunized with rGD-SX-5U2 or DMEM were subjected to HP-PRRSV SD-YL1712 or NADC30-like PRRSV SX-YL1806 challenge at 42 dpv. Sera were collected at the indicated time points. All the piglets were euthanized at 63 dpv. **B**–**G** Temperature, average daily gain rate (ADG), PRRSV-N antibody level, virus-neutralizing antibody titre and virus load of the animal experiment. (B) Rectal temperature of pigs recorded every day. **C** Average daily gain rate of piglets measured weekly. **D** PRRSV-N protein-specific antibodies were measured weekly by a Porcine Reproductive and Respiratory Syndrome Virus AB Elisa Kit (JNT, China) according to the manufacturer’s instructions. **E** Viral neutralization properties of immune sera from 42 dpv. IFA was used to confirm the CPE induced by PRRSV. The absence of CPE at a 1:8 dilution was considered positive for the presence of PRRSV neutralization. Real-time qPCR was used to determine the viral load in the lung tissue (**G**) and serum (**F**). A recombinant plasmid containing the ORF7 gene of PRRSV was used to construct a standard curve. The number of PRRSV RNA copies was calculated according to the standard curve.

To evaluate the cross-protection capacity of the rescued chimeric PRRSV rGD-SX-5U2, vaccination and challenge strategies were designed as illustrated in Figure 4A and Table 2. After challenge at 42 dpv, piglets in groups A and B presented significant clinical manifestations, including diarrhoea from 43 dpv for approximately one week, anorexia from 45 dpv for approximately two weeks, and coughing and sneezing from 46 dpv for one week or more. Among them, the symptoms of the piglets in group A were more severe and lasted longer. After immunization at 0 dpv, piglets in groups C and D presented mild diarrhoea symptoms but quickly returned to normal after 3 days. After challenge at 42 dpv, the piglets in group C presented no obvious clinical symptoms; the piglets in group D presented transient diarrhoea symptoms but returned to normal after 2 days, with no other abnormal manifestations. The piglets in group E showed no abnormalities throughout the experiment.

The rectal temperature of each piglet was measured daily after immunization at 0 dpv. After immunization with PRRSV rGD-SX-5U2 at 0 dpv, the rectal temperature of the piglets in groups C and D temporarily increased, among which those in group C appeared at 2 dpv, 5 dpv, 6 dpv, 7 dpv, and 10 dpv, and those in group D appeared at 6 dpv, 7 dpv, 9 dpv, 10 dpv, and 11 dpv. No fever was observed in the piglets of group A, group B, or group E before 42 dpv. After being challenged with HP-PRRSV SD-YL1712 or NADC30-like PRRSV SX-YL1806 at 42 dpv, the rectal temperature of piglets in groups A and B increased significantly, among which the piglets in group A had a fever state for 11 days, with a peak temperature of 40.63 °C, which occurred at 53 dpv. The piglets in group B had a fever state that lasted for 12 days, with a peak temperature of 40.9 °C at 47 dpv. Conversely, after being challenged at 42 dpv, the rectal temperature of the piglets in groups C and D remained normal, and there were no symptoms of fever. The rectal temperatures of mock-infected pigs in group E were always lower than 40 °C (Figure 4B).

The average daily gain (ADG) of piglets in different groups was recorded weekly after challenge with HP-PRRSV SD-YL1712 or NADC30-like PRRSV SX-YL1806 at 42 dpv. At 49 dpv, there was no significant difference in ADG levels between groups C and A (*P* > 0.5), both of which were significantly lower than those in group E (*P* < 0.01). Conversely, the ADG level in group D was significantly higher than that in group B (*P* < 0.01) and there was no significant difference between group D and group E (*P* > 0.05). At 56 dpv, the ADG levels of both group C and group D were significantly higher than those of group A and group B (*P* < 0.01), and there was no significant difference from that of group E (*P* > 0.05). At 63 dpv, the ADG level of group C was significantly higher than that of group A (*P* < 0.01). In addition, there was no significant difference in ADG levels between group C and group D compared with group E (*P* > 0.05) (Figure 4C).

The serum samples of pigs in each group were collected at different time points. After immunization with chimeric PRRSV rGD-SX-5U2, the levels of antibodies against the PRRSV-N protein in piglets in groups C and D gradually increased, and seroconversion (S/P > 0.4) occurred at 21 dpv, after which the antibodies gradually increased. After being challenged with HP-PRRSV SD-YL1712 or NADC30-like PRRSV SX-YL1806 at 42 dpv, the levels of antibodies against the PRRSV N protein in groups C and D initially decreased slightly but then increased significantly, peaking at 1.60 and 1.88, respectively (Figure 4D).

To explore the degree of virus neutralization induced by chimeric PRRSV rGD-SX-5U2, serum samples collected at 42 dpv from groups C and D (*n* = 10) were used for a virus neutralization (SVN) test. The results indicated that 3 piglets in these two groups exhibited neutralizing activity against HP-PRRSV SD-YL1712 (≥ 1:8) and that 3 piglets presented neutralizing activity against NADC30-like PRRSV SX-YL1806 (≥ 1:8) (Figure 4E).

Serum samples from pigs in each group were collected at 4, 7, 14, 21, 28, 35, 42, 49, 56, and 63 dpv for analysis of viremia using RT-qPCR. The results revealed that after challenge at 42 dpv, the piglets in group C presented significantly fewer serum viral copies than did those in group A. At 63 dpv, the piglets in group A still presented high viremia, whereas the piglets in group C presented reduced serum viral copies. Similarly, the piglets in group D had significantly lower viremia than those in group B did. By 63 dpv, the PRRSV viremia of the piglets in group D had been reduced to extremely low levels (Figure 4F).

All the piglets were euthanized at the end of the experiment, and the lung tissues from each piglet were collected to determine the PRRSV load. The results indicated that PRRSV could be detected in all challenged pigs. The viral loads in the lung tissue of piglets in groups A and B were greater, whereas no PRRSV was detected in group E. Moreover, compared with those in group A, the viral loads in the lung tissue of piglets in group C were significantly lower (*P* < 0.001). Similarly, the viral load in the lung tissue of piglets in group D was significantly lower than that in group B (*P* < 0.001) (Figure 4G).

### The chimeric PRRSV rGD-SX-5U2 provided protection for lung tissues

**Figure 5:**
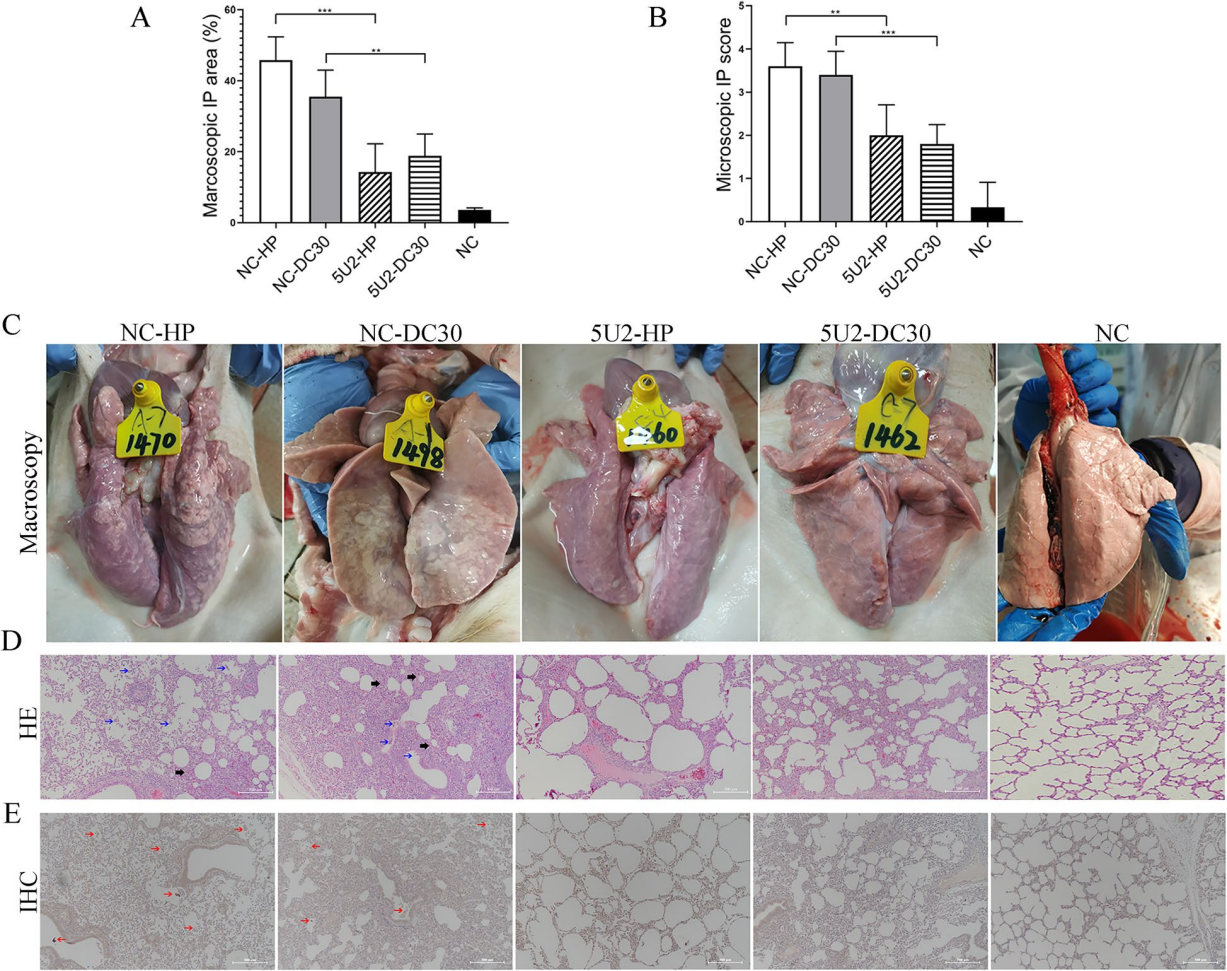
**Macroscopic and microscopic tissue lesions and immunohistochemistry results for infected pigs**. All pigs were euthanized for autopsy at 63 dpv, and their lung tissues were collected and analysed. Macroscopic and microscopic tissue lesions and immunohistochemistry analysis of infected pigs. **A**, **C** The macroscopic lesions and scores of the lungs were estimated by assigning a number to each lobe to reflect the approximate percentage of the entire lung represented by that lobe. **B**, **D** Pig lung slices stained with haematoxylin and eosin (HE). The degree and severity of interstitial pneumonia were taken into account when the microscopic lesion and score of the lung tissues were calculated: 0, no lesion; 1, mild/focal; 2, moderate/multifocal; 3, moderate/diffuse; and 4, severe/diffuse. Three pathologists evaluated the lung lesions, both microscopically and macroscopically, via blind examinations. **E** Detection of the PRRSV-N protein in the lung tissue of pigs via an IHC assay. The blue arrows indicate thickening of the alveolar septum and a decrease in the alveolar space; the black arrows indicate infiltration of inflammatory cells; and the red arrows indicate positive signs of PRRSV.

During the autopsy, lung injury was observed, and the lung tissues were collected for HE and IHC testing. The results revealed that the lungs of piglets in groups A and B presented obvious interstitial pneumonia, mainly pulmonary consolidation, oedema, and haemorrhage, among which there were more severe macroscopic lesions of the lungs in group A than in group B. Moreover, the number of macroscopic lesions of the lungs in group C was significantly lower than that in group A, and similarly, the number of macroscopic lesions of the lungs in group D were significantly greater than that in group B. No obvious macroscopic lesions of the lungs were observed in group E (Figures 5A–C).

There were obvious microscopic lesions in groups A and B, including thickening of the alveolar septum (black arrows), shrinkage of the alveolar space, and inflammatory cell infiltration (blue arrows). In contrast, compared with those in group A, the microscopic lesions of the lungs in group C were significantly reduced, and the microscopic lesions of the lungs in group D were also alleviated compared with those in group B. No obvious microscopic lesions of the lungs were found in group E (Figures 5B–D).

Moreover, IHC staining was used to confirm the presence of PRRSV in the lung tissues. The results revealed many positive signals for the PRRSV-N protein in the lung tissues of piglets in groups A and B (red arrows). Similarly, the positive signals of PRRSV-N protein in the lung tissue of piglets in groups C and D were significantly reduced, and there was no abnormal signal in group E (Figure 5E).

These results suggested that immunization with the chimeric PRRSV rGD-SX-5U2 conferred cross-protection against both HP-PRRSV and NADC30-like PRRSV.

## Discussion

Vaccination represents a crucial strategy for the prevention and control of PRRSV, particularly in the wake of the HP-PRRSV outbreak in 2006. However, as PRRSV continues to mutate, the genomic homology between different PRRSV strains gradually decreases, and PRRSV diversity gradually increases [14]. Currently, at least nine distinct commercial PRRSV vaccines are available in China. Furthermore, these commercial vaccines are all based on classic PRRSV or HP-PRRSV strains [22, 26]. However, NADC30-like PRRSV exhibits low genomic homology with both classical PRRSV and HP-PRRSV strains, rendering the production of effective cross-protective vaccines against this virus challenging [44]. Therefore, the development of a new vaccine that can provide good protection against NADC30-like PRRSV is highly important for the prevention and control of PRRSV.

The chimeric virus strains constructed on the basis of reverse genetic manipulation technology have made interesting attempts to develop new vaccines in recent years [45–48]. The PRRSV SP MLV vaccine, which was obtained in 2018, is a recombinant vaccine strain formed by the chimerism of classic PRRSV and HP-PRRSV. Furthermore, Chen et al. synthesized the conserved genes of PRRSV ORF2‒ORF6 and embedded them into the HP‒PRRSV strain to construct the chimeric PRRSV rJS‒ORF2‒6‒CON, and the results demonstrated that it provided robust protection against NADC30 PRRSV [26]. Furthermore, Gao et al. generated the chimeric virus strain rPRRSV-E2 by utilizing the commercial vaccine strain HuN4-F112 as the backbone and embedding the E2 gene of classical swine fever virus (CSFV). The chimeric rPRRSV-E2 demonstrated efficacy in providing protection against both PRRSV and CSFV [49–51]. In this study, we used the commercial vaccine strain GD as the backbone and embedded several genes of NADC30-like PRRSV to construct the chimeric PRRSV rGD-SX-5U2, which provides protection against both HP-PRRSV and NADC30-like PRRSV. The PRRSV GD strain was selected as the backbone owing to its status as a vaccine strain and its safety profile in piglets (Figure 2A).

PRRSV exhibits a strict host and cellular tropism [32]. However, an intriguing phenomenon has been observed whereby the majority of NADC30-like PRRSV strains lose their tropism to MARC-145 cells [34, 35]. Studies have been conducted to explore the mechanisms affecting PRRSV tropism. A chimeric virus strain was constructed by inserting the PRRSV ORF5 and ORF6 genes into the corresponding locations of equine arteritis virus (EAV). However, this chimeric strain did not acquire tropism to MARC-145 cells, which suggests that the key viral genes that determine PRRSV tropism are not the ORF5‒ORF6 genes [52]. Zhang et al. replaced the ORF2a-ORF3 genes of the PRRSV MY-376 (HP-PRRSV, which cannot infect MARC-145 cells) strain with the ORF2a-ORF3 genes of the PRRSV HuN4-F112 (HP-PRRSV, which can infect MARC-145 cells) strain to construct a chimeric PRRSV vMY-376-TORF2a-ORF3. This chimeric PRRSV can infect MARC-145 cells, indicating that ORF2a‒ORF3 may be responsible for the tropism of HP-PRRSV to MARC-145 cells [53]. MARC-145 cells represent a crucial platform for the development of PRRSV vaccines. In this study, to obtain a chimeric PRRSV strain with cell tropism to MARC-145 cells, the ORF2a, ORF3, and ORF4 genes of NADC30-like PRRSV SX-YL1806, which had lost cell tropism to MARC-145, were not chosen to assemble into the PRRSV GD to construct the chimeric PRRSV rGD-SX-5U2, as these genes may interact with the core receptor CD163 of PRRSV. Instead, the ORF5, ORF6, and ORF7 genes of NADC30-like PRRSV SX-YL1806 were introduced into PRRSV GD. The results of the growth curve demonstrated that rGD-SX-5U2 retained its ability to infect MARC-145 cells and exhibited a high viral titre (10^5.625^/mL) (Figure 3D). These results indicated that the ORF5-ORF7 and NSP2 genes of NADC30-like PRRSV did not determine its tropism to MARC-145 cells.

The results of the animal experiments demonstrated that the chimeric PRRSV rGD-SX-5U2 could provide protection against both HP-PRRSV and NADC30-like PRRSV. After being attacked at 42 dpv, piglets in the rGD-SX-5U2-immunized group (groups C and D) presented minimal clinical symptoms and maintained a normal body temperature; ADG levels were not significantly different from those in the NC group (group E), and viremia decreased to very low levels at 63 dpv (Figures 4B, C, F). Moreover, the viral load in the lung tissue was significantly lower in both immune groups (groups C and D) than in the unimmunized groups (groups A and B) (Figure 4G). In contrast, piglets in the unimmunized group presented clinical symptoms, including coughing, sneezing, diarrhoea, and prolonged fever, following infection with HP-PRRSV or NADC30-like PRRSV at 42 dpv. The peak temperatures were recorded at 40.63 °C and 40.9 °C. Furthermore, there was a notable decline in average daily gain (ADG) levels and a high level of viremia (Figures 4B, C, F). In addition, the lung tissue lesions were consistent with the above indicators, and piglets immunized with rGD-SX-5U2 presented mild lung tissue lesions after being attacked by HP-PRRSV or NADC30-like, whereas unimmunized piglets presented severe lung tissue lesions (Figure 5). These results suggest that immunization with the chimeric PRRSV rGD-SX-5U2 confers effective cross-protection against both HP-PRRSV and NADC30-like PRRSV.

Further analysis revealed that the chimeric PRRSV rGD-SX-5U2 continued to exert some negative effects on piglets. First, the rectal temperature of piglets immunized with rGD-SX-5U2 slightly increased. Group C exhibited slight increases in temperature (over 40 ℃) at 2 dpv, 5 dpv, 6 dpv, 7 dpv, and 10 dpv, whereas group D exhibited similar trends at 6 dpv, 7 dpv, 9 dpv, 10 dpv, and 11 dpv. However, the fever subsided by 12 dpv (Figure 4B). Furthermore, immunization with rGD-SX-5U2 also affected the ADG of piglets, especially before 21 dpv (Figure 4C). In addition, immunization with the rGD-SX-5U2 strain also increased the viremia of piglets, especially at 21 dpv, and the viremia of piglets in Groups C and D reached a relatively high level (Figure 4F). These results indicate that the chimeric PRRSV rGD-SX-5U2 may retain low pathogenicity in piglets. Chen et al. demonstrated that the NSP2 gene of HP-PRRSV plays a crucial role in both its in vitro infection efficiency and virulence. This was evidenced by the observed effects on the survival rate, temperature, viremia, lung score, and tissue score [54]. Similarly, Kong et al. reported that the NSP2 genes of HP-PRRSV are critical virulence regulators and highlighted the importance of NSP2 genetic variation in modulating PRRSV virulence and persistence via immune modulation [55]. In this study, chimeric PRRSV rGD-SX-5U2 was derived from the NADC30-like PRRSV (ORF5, ORF6, ORF7, and NSP2 genes) and the MLV-vaccine strain (other genes). It was postulated that there may be unidentified genes among the ORF5, ORF6, ORF7, and NSP2 genes that affect the pathogenicity of NADC30-like PRRSV, such as the NSP2 gene. However, further research is needed to validate this hypothesis.

Chen et al. synthesized a consensus sequence of PRRSV2 ORF2-6 genes, which encode all the envelope proteins of PRRSV. This approach was used to generate a chimeric virus, which was found to induce broadly neutralizing antibodies against the NADC30-like isolate [26]. Su et al. reported that NSP2 and GP5-M are associated with limited neutralization reactivity between the heterologous strains HP-PRRSV and LP-PRRSV (low-pathogenic PRRSV strains) [56]. In this study, to increase the capacity of the chimeric PRRSV strain to elicit increased levels of neutralizing antibodies, particularly those with broad-spectrum reactivity against NADC30-like PRRSV, chimeric PRRSV was constructed by replacing the NSP2 gene alongside the ORF5, ORF6, and ORF7 genes from NADC30-like PRRSV. The results demonstrated that immunization with chimeric PRRSV rGD-SX-5U2 provided robust protection against both HP-PRRSV and NADC30-like PRRSV. However, the level of neutralizing antibodies against PRRSV remained low at 42 days post-vaccination, with only three piglets out of ten immunized piglets showing neutralizing activity against homologous HP-PRRSV or heterologous NADC30-like PRRSV (Figure 4E). Furthermore, it appears that the NSP2, ORF5, ORF6, and ORF7 genes of the NADC30-like PRRSV did not markedly enhance the neutralizing antibodies of the chimeric PRRSV. Consequently, we speculate that other genes may be involved in the neutralizing activity of NADC30-like PRRSV, perhaps among the ORF2-ORF4 genes, but more research is needed. In addition, factors other than neutralizing antibodies may be involved in the protective effect of the chimeric PRRSV rGD-SX-5U2 against the homologous strain HP-PRRSV and the heterologous strain NADC30-like PRRSV, such as cellular immunity, since the neutralizing antibodies induced by rGD-SX-5U2 were not significantly increased, but further research is needed to confirm this hypothesis.

Notably, the genomic homology between PRRSV vaccine strains and epidemic strains is an important factor affecting the protective effect of the PRRSV vaccine, but other factors may still play a role. Our previous studies suggested that antibody-dependent enhancement (ADE) may influence the protective effect of PRRSV MLV vaccines against heterologous PRRSV strains [28]. In addition, the development of reasonable immunization programs involving different vaccines is also important [57–59]. Therefore, to improve the homology of the PRRSV vaccine strain and epidemic strain, constructing a vaccine without ADE and optimizing the immunization program may also be important methods to improve the efficacy of the PRRSV vaccine, and more research is needed.

In conclusion, a chimeric PRRSV strain of HP-PRRSV and NADC30 PRRSV, rGD-SX-5U2, was constructed in this study. The chimeric PRRSV strain rGD-SX-5U2 was capable of infecting MARC-145 cells. The results of the pig inoculation and challenge studies demonstrated that rGD-SX-5U2 conferred effective cross-protection against both HP-PRRSV and NADC30-like PRRSV. This was evidenced by the absence of fever following challenge, improvements in ADG levels, reductions in viremia and lung tissue lesions, and so forth. The chimeric rGD-SX-5U2 may be a promising candidate for the development of a novel, broadly protective vaccine. Furthermore, this chimeric PRRSV can be employed in basic research on PRRSV, including the identification of novel pathogenic viral genes or the neutralization of epitopes.

## Data Availability

All the data generated during the current study are included in the manuscript.
